# *Fusarium solani* species complex infection in elasmobranchs: A case report for rough-tail stingray with valid antifungal therapy

**DOI:** 10.1016/j.mmcr.2021.02.002

**Published:** 2021-02-18

**Authors:** Li-Hang Hsu, Chen-Yi Su, Pei-Lun Sun, Ying-Lien Chen

**Affiliations:** a Department of Plant Pathology and Microbiology, National Taiwan University, No. 1, Sec. 4, Roosevelt Road, Taipei 10617, Taiwan; b Farglory Ocean Park, No. 189, Fude Road, Hualien 97449, Taiwan; c Institute of Veterinary Medicine, School of Veterinary Medicine, National Taiwan University, No. 1, Sec. 4, Roosevelt Road, Taipei 10617, Taiwan; d Department of Dermatology and Research Laboratory of Medical Mycology, Chang Gung Memorial Hospital, Linkou Branch, No. 5, Fushin St., Taoyuan 33305, Taiwan; e College of Medicine, Chang Gung University, No.259, Wenhua 1st Rd., Taoyuan, 33302, Taiwan

**Keywords:** Fusarium, FSSC, Fungal infection

## Abstract

*Fusarium* species are common plant and animal pathogens. For humans, there are two dominant species complexes, *F. solani* species complex (FSSC) and *F. oxysporum* species complex (FOSC), which both infect immunocompromised individuals. However, there are few reports related to elasmobranchs infected by *Fusarium* species. In this study, we report a case of a rough-tail stingray from an ocean park infected by FSSC diagnosed using histopathology and microscopic observation, with morphological characteristics and molecular techniques used to identify the pathogen. Histopathology showed fungal hyphae invading stingray tissues, while micro/macroconidia were found under the microscope. We identified this pathogen as FSSC 12 through phylogenetic analysis using internal transcribed spacer (ITS) and elongation factor 1-alpha (EF1-α) sequences. Furthermore, we report that application of voriconazole (orally) and terbinafine (topically) constituted an effective therapy, curing the stingray.

## 1 Introduction

The genus *Fusarium* can cause multiple fungal infections in plants and animals worldwide, resulting in reduced crop production, fatal mycotoxins or diseased individuals [[1], [2], [3]]. In humans, *F. solani* species complex (FSSC) and *F. oxysporum* species complex (FOSC) are the most prevalent and can cause superficial or invasive fusariosis [4]. Fusarium infections in elasmobranchs are rare, and the first FSSC infection was reported in a black spotted stingray in 2015 [5]. FSSC infections can cause cutaneous lesions that turn to ulcers on the skin surface and lead to death of infected animals [5].

However, literature documenting fungal infections in cartilaginous or ornamental fish is rare, probably as therapeutic drugs are limited and expensive. In this report, we described a rough-tail stingray from Farglory Ocean Park (Hualien, Taiwan [E121°36′10″, N23°54′04"]) infected by *F. solani* species complex. The diseased stingray was cured by applying antifungal drugs voriconazole (orally) and terbinafine (topically).

## 2 Case

A white discolored lesion was observed on the dorsal aspect of a rough-tail stingray (*Dasyatis centroura*) in 2016. The stingray was born at Farglory Ocean Park (Hualien, Taiwan) in March 2016 and kept in an area of 5.8 m × 3.1 m × 0.75 m, which was connected to a tank of 750,000 L. Water quality parameters were: temperature 24.7–26 °C, salinity 33.3–33.7 parts per thousand, pH 7.5–8.3, and oxygen 6.6–6.8 mg L^-1^. The stingray was fed capelin and horse mackerel. To prevent contagious infection, the diseased stingray was quarantined immediately in a tank of 1300 L, and the water quality parameters were: temperature 23.7–26 °C, salinity 33.1–33.9 parts per thousand, pH 8.1–8.7, and oxygen 6.4–6.9 mg L^-1^.

**Fig. 1:**
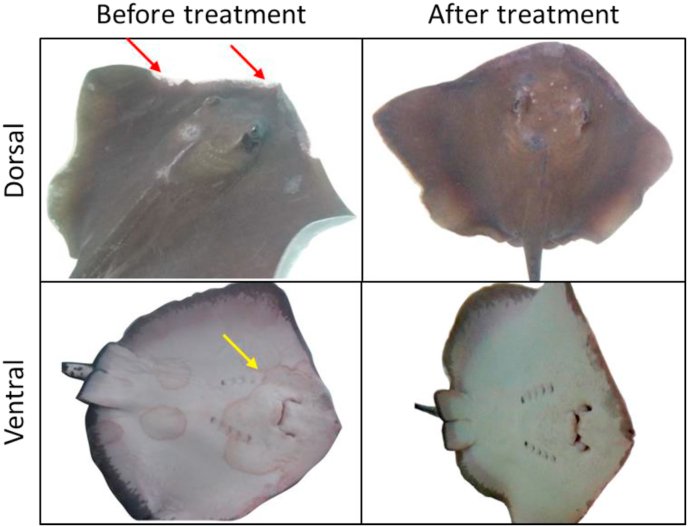
**Combination of voriconazole and terbinafine is therapeutic to rough-tail stingray infected with *Fusarium solani*.** Before drug application, white and discolored lesions were seen on the dorsal aspect of the snout, and the lesions eventually turned to ulceration (red arrowheads). On the ventral side, cutaneous ulcers with light tan patches and red margins appeared, especially around the mouth and gill slits (yellow arrowhead). After systemic voriconazole and topical terbinafine antifungal therapy, the white lesions on the dorsal side decreased, and the patches on the ventral side disappeared after 15 weeks. (For interpretation of the references to color in this figure legend, the reader is referred to the Web version of this article.)

For disease symptom observation, skin scraping was performed in mid-May 2016, and a large number of hyphae were noted under a light microscope. Consequently, for immediate therapy, 10% iodine solution, methylene blue, merbromin solution and salicylic acid were applied topically, but to no avail. The situation got worse in early June when the symptoms became cutaneous ulcers on the ventral side, and pink-to-light tan patches with reddish rims were seen, especially around the mouth and gill slits (Fig. 1). Through skin scraping and microscopic examination, fungal hyphae were noted on ventral cutaneous lesions. Biopsy was performed on July 1st, 2016. Tricaine methanesulfonate (MS-222) 50 ppm was used for anesthesia, and the biopsy punch was 4 mm in diameter and two specimens were obtained from the diseased stingray. Each biopsied tissue was subdivided into two portions. The major portion was fixed in 10% neutral buffered formalin and submitted to Chang Gung Memorial Hospital (Linkou, Taoyuan, Taiwan) for histopathologic diagnosis, and the minor portion was preserved in the transport medium and submitted to the department of Plant Pathology and Microbiology, National Taiwan University (Taipei, Taiwan) for fungal identification and antifungal susceptibility test.

### 2.1 Histopathology and pathogen identification

**Fig. 2:**
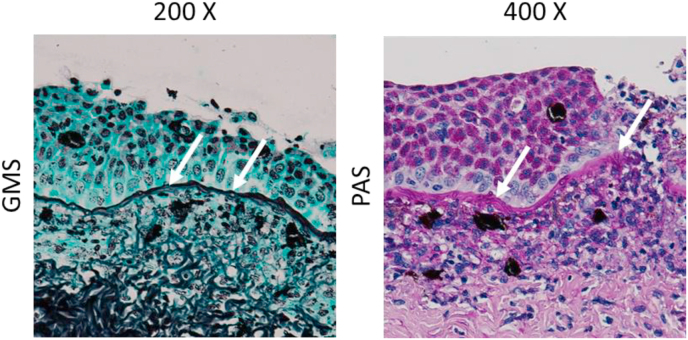
**Histopathology analysis of the infected rough-tail stingray.** Tissue samples of infected stingray were fixed in 10% neutral buffered formalin, sectioned at 4 μm, and stained with Gomori methenamine silver (GMS) or periodic acid–Schiff (PAS). White arrows represent invasive hyphae of *F. solani*.

All tissue samples were fixed in 10% neutral buffered formalin, sectioned at 4 μm, and stained with periodic acid–Schiff (PAS) and Gomori methenamine silver (GMS). Upon histopathological examination, the cutaneous lesion revealed a moderate to severe, chronic, multifocal to coalescing, and ulcerative dermatitis, and the hypodermis was infiltrated with inflammatory cells (granulocytes and phagocytes). Branching, and septate fungal hyphae existed in the dermis and the junction between the epidermis and dermis (Fig. 2).

**Fig. 3:**
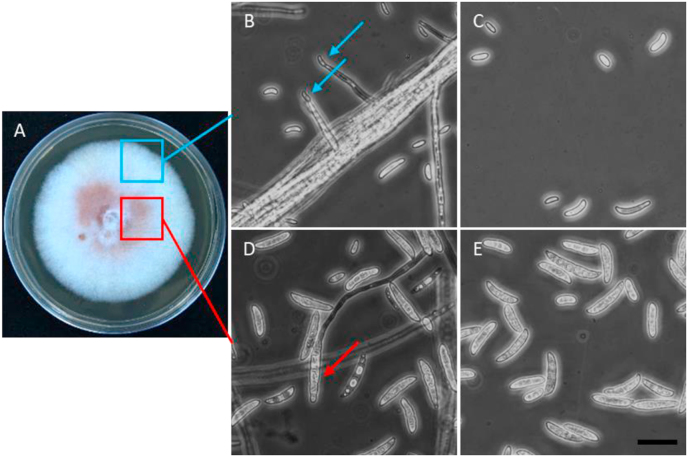
**Morphology of *Fusarium solani* LHS13 isolated from the infected rough-tail stingray.** (A) The *F. solani* colony on PDA medium. An agar disc with fungal culture was placed on a PDA agar plate, and incubated at 28 °C for 7 days. (B)&(C) Microscopic morphology of *F. solani* obtained from the colony edge (blue square). The micro-conidiophore was signed by blue arrows. (D)&(E) Microscopic morphology of *F. solani* obtained from colony center (red square). The macro-conidiophore is indicated by the red arrow. Scale bar = 20 μm. (For interpretation of the references to color in this figure legend, the reader is referred to the Web version of this article.)

The isolated pathogen was incubated on the PDA agar plate for morphology observation. The colony morphology showed white and flat hyphae with a little red-to-purple pigment in the center of the colony (Fig. 3A). On the other hand, through the ITS and EF1-α sequences blast and analyses, we found that this pathogen belonged to the *Fusarium solani* species complex, and named *F. solani* LHS13 [6,7]. To further characterize its microscopic structure, parts of colony were streaked out and observed with a light microscope. It was noted that *F. solani* produced two types of conidia: macroconidia and microconidia like most of *Fusarium* species do [8]. The macroconidia were straight with a rounded apical or basal cell, comprised of 6–7 cells, and present near the central colony, while the microconidia were elliptical, comprised of 1–2 cell(s), and mostly found at the edge of colony (Fig. 3B–E).

### 2.2 Phylogenetic analysis

**Fig. 4:**
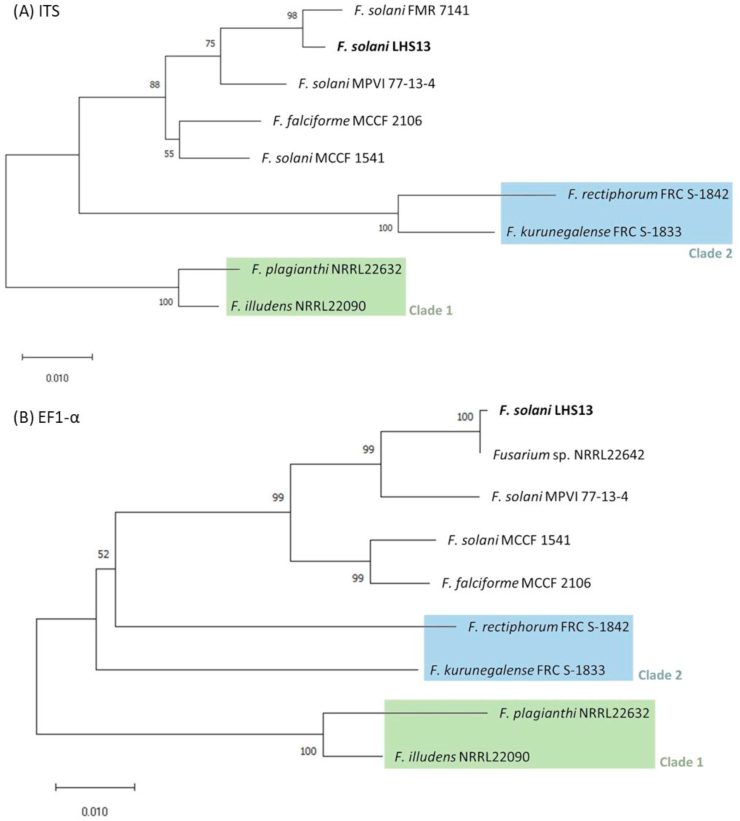
**Phylogenetic analysis of *Fusarium solani* LHS13 isolated from the infected rough-tail stingray by using (A) internal transcribed spacer (ITS) and (B) elongation factor 1 alpha (EF1-α) sequences.** The phylogenetic analyses were performed by MEGA X 10.1.7 software with the neighbor-joining method and inferred from ITS and EF1-α sequence data respectively. The phylogenetic trees were rooted using clade 1 of the FSSC. The percentage of replicate trees in which the associated taxa clustered together in the bootstrap test (1000 replicates) are shown next to the branches. The tree is drawn to scale, with branch lengths in the same units as those of the evolutionary distances used to infer the phylogenetic tree. The evolutionary distances were computed using the Maximum Composite Likelihood method and are in the units of the number of base substitutions per site.

The ITS or EF1-α DNA fragments were amplified with primer pair ITS5/ITS4 (GGAAGTAAAAGTCGTAACAAGG/TCCTCCGCTTATTGATATGC) or EF-1/EF-2 (ATGGGTAAGGARGACAAGAC/GGARGTACCAGTSATCATGTT) respectively, sequenced (MissionBiotech, Taiwan) and searched using the algorithm of basic local alignment search tool (BLAST) in GenBank from National Center for Biotechnology Information (NCBI, https://blast.ncbi.nlm.nih.gov/Blast.cgi) and from the *Fusarium* Multi Locus Sequence Typing Database (MLST, http://www.wi.knaw.nl/Fusarium/). Results showed that the pathogen isolated from infected stingray possessed 99.8% identity with *F. solani* NRRL46705 FSSC12 strain FMR7141 (GenBank: AM412637) in ITS sequence and 100% identity with *Fusarium* sp. NRRL 22642 FSSC12 (GenBank: DQ246844) in EF1-α sequence. The phylogenetic analyses were then schemed by MEGA X 10.1.7 software using the neighbor-joining method, and inferred from ITS or EF1-α sequence data respectively (Table 1) (Fig. 4). Sequences were aligned by MUSCLE algorithm and the phylogenetic trees were rooted using clade 1 of the FSSC [5,9].

**Table 1:** *Fusarium* species and DNA sequence information used in this study.

***Fusarium* species**	**strain**	**Source**		**Reference**
*F. solani*	LHS13	Stingray		This study
*F. falciforme*	MCCF 2106	Clinical (from keratitis patient)		This study
*F. solani*	MCCF 1541	Clinical (from onychomycosis patient)		This study
*F. solani*	MPVI 77-13-4	Plant		[18]
***Fusarium* species**	**strain**	**DNA accession number in NCBI**		**Reference**
		**ITS**	**EF1-α**	
*F. rectiphorum*	FRC S-1842	JF433043	JF433026	[5,9]
*F. kurunegalense*	FRC S-1833	JF433036	DQ247511	
*F. plagianthi*	NRRL22632	AF178417	AF178354	
*F. illudens*	NRRL22090	AF178393	AF178326	
*F. solani*	NRRL46705/FMR7141	AM412637	–	[14]
*Fusarium sp.*	NRRL 22642	–	DQ246844	[15]

### 2.3 Antifungal susceptibility test

Antifungal susceptibility test was performed following the Clinical and Laboratory Standards Institute guideline M38-A2 [10]. We found that voriconazole and terbinafine can inhibit the growth of *F. solani* LHS13 at 8 μg/mL for both minimal inhibitory concentration (MIC) and minimal fungicidal concentration (MFC).

### 2.4 Antifungal therapies

Antifungal therapy (Table 2) was started with itraconazole (Sporanox, Janssen, Belgium) on June 24th, 2016, and topical treatment with a combination of terbinafine (Lamisil, Swiss) and dimethyl sulfoxide (DMSO 90% gel) was performed from July 5th, 2016. Oral itraconazole was replaced with voriconazole (VFend, Pfizer, America) at 3 mg/kg from July 21st, 2016, because the disease exacerbated. Although the systemic and topical antifungal drugs were given, the cutaneous lesions still expanded rapidly. Thus, the dosage of voriconazole was then adjusted to 4 mg/kg from August 18th, 2016 while topical treatment of terbinafine continued, and an antibiotic therapy using enrofloxacin at 10 mg/kg (Baytril, Bayer, Germany) was initiated in order to prevent secondary infections (Table 2).

**Table 2:** Administration of antimicrobial drugs to a stingray infected by *F. solani*.

Date	Drug	Dosage and application	Comments
2016/06/24	Itraconazole (Sporanox)	5 mg/kg, *PO SID*	
2016/07/05	Itraconazole	5 mg/kg, *PO SID*	
	Terbinafine (Lamisil)	5 g, topical	
2016/07/21	Voriconazole (VFend)	3 mg/kg, *PO SID*	Lesions still extended rapidly and disease exacerbated.
	Terbinafine	5 g, topical	
2016/08/18	Voriconazole	4 mg/kg, *PO SID*	Epidermis began to recover from ulcers in early September 2016.
	Enrofloxacin (Baytril)	10 mg/kg, *PO SID*	
	Terbinafine	5 g, topical	
*PO*: by mouth (*per os*); *SID*: once a day (*semel in die*)			

In early September 2016, the epidermis of rough-tail stingray gradually changed to a white discolored lesion in the dorsal aspect and fewer hyphae were found. Thus, systemic oral voriconazole and topical terbinafine treatments were continued for one additional month, and eventually no fungus was noted in the same area from October 13th, 2016. Oral voriconazole and topical terbinafine was discontinued on November 4th, 2016 since the stingray no longer showed any symptoms.

## 3 Discussion

In *Fusarium oxysporum* species complex (FOSC), there are chromosomes called lineage-specific (LS) chromosomes, which contain genes associated to host specificity, and these LS chromosomes are different in FOSC infecting plants and humans [11]. In FSSC, there are some additional chromosomes other than core chromosomes, which are similar to LS chromosomes and are called conditionally dispensable (CD) supernumerary chromosomes. Features of these CD supernumerary chromosomes are also associated with host specificity [12]. We identified the pathogen of the diseased stingray through morphological observation and phylogenetic analysis using ITS and EF1-α sequencing, and found that the pathogen belonged to FSSC clade 3, which was comprised of most *F. solani* strains [13]. In addition, sequence BLAST from NCBI or MLST showed that this pathogen came from FSSC 12, which was similar to the results described in 2015 [5], and the most related strains FMR7141 and NRRL 22642 were isolated from aquarium sand [14] and prawn [15], respectively. Consequently, it could be inferred that the captive environment possibly harbors *Fusarium* which could infect aquatic organisms. However, whether FSSC 12 was specific to its host, elasmobranchs, remains unclear.

In terms of therapeutic cases related to elasmobranchs before 2020, there was a case using voriconazole to cure *Fusarium* infection of bonnethead sharks [16], and there was no report of successful terbinafine application. In addition, some cases showing the recovering from bacterial infection by using an antibiotic enrofloxacin were also included in an elasmobranchs husbandry manual [17]. Using our therapeutic approach, the colonization of the pathogen was reduced through the application of voriconazole and terbinafine, and secondary infection with bacteria invading through the epidermis wounds was prevented by applying enrofloxacin. There was less information documenting topical dosage of antifungal drug applied on elasmobranchs, so the strategies of drug application in this case might be assessed as a potential guideline for stingrays infected by *F. solani*.

Cases of marine creatures infected by fungal pathogens have seldom been investigated, and few reports mention elasmobranchs like sharks or stingrays, which may be because of the high cost of health care and the inconvenience of drug application. Here, we have presented a successful therapeutic strategy in a rough-tail stingray infected by *F. solani* in an artificial environment.

## Ethical statement

All experiments including tissue collection and drug administration in this study complied with ethical standards, and the diseased animal (*i.e.* stingray) observed in this case report was well cared and quarantined in appropriate environment.

## Consent

Written informed consent was obtained from the patient or legal guardian(s) for publication of this case report and accompanying images. A copy of the written consent is available for review by the Editor-in-Chief of this journal on request.

## Funding sources

This work was financially supported by grant 107-2320-B-002-061-MY3 from the 10.13039/501100004663Ministry of Science and Technology in Taiwan.

## Declaration of competing interest

There are no conflicts of interest.

## References

[1] Figueroa M., Hammond-Kosack K.E., Solomon P.S. (2018). A review of wheat diseases-a field perspective. Mol. Plant Pathol..

[2] Summerell B.A. (2019). Resolving *Fusarium*: current status of the genus. Annu. Rev. Phytopathol..

[3] Moretti A., Logrieco A.F., Mycotoxins A. Susca (2017). An underhand food problem. Methods Mol. Biol..

[4] van Diepeningen A.D., Al-Hatmi A.M.S., Brankovics B., de Hoog G.S. (2014). Taxonomy and clinical spectra of *Fusarium* species: where do we stand in 2014?. Curr. Clin. Microbiol. Rep..

[5] Fernando N., Hui S.W., Tsang C.C., Leung S.Y., Ngan A.H., Leung R.W. (2015). Fatal *Fusarium solani* species complex infections in elasmobranchs: the first case report for black spotted stingray (*Taeniura melanopsila*) and a literature review. Mycoses.

[6] O'Donnell K., Kistler H.C., Cigelnik E., Ploetz R.C. (1998). Multiple evolutionary origins of the fungus causing Panama disease of banana: concordant evidence from nuclear and mitochondrial gene genealogies. Proc. Natl. Acad. Sci. U. S. A..

[7] White T.J., Bruns T., Lee S., Taylor J., Innis M.A., Gelfand D.H., Sninsky J.J., White T.J. (1990). Amplification and direct sequencing of fungal ribosomal RNA genes for phylogenetics. PCR Protocols: a Guide to Methods and Applications.

[8] Leslie J.F., Summerell B.A. (2006). Species Descriptions.

[9] Sandoval-Denis M., Lombard L., Crous P.W. (2019). Back to the roots: a reappraisal of *Neocosmospora*. Persoonia.

[10] CLSI (2008). Reference Method for Broth Dilution Antifungal Susceptibility Testing of Filamentous Fungi; Approved Standard.

[11] Ma L.-J., van der Does H.C., Borkovich K.A., Coleman J.J., Daboussi M.-J., Di Pietro A. (2010). Comparative genomics reveals mobile pathogenicity chromosomes in *Fusarium*. Nature.

[12] Waalwijk C., Taga M., Zheng S.L., Proctor R.H., Vaughan M.M., O'Donnell K. (2018). Karyotype evolution in *Fusarium*. IMA Fungus.

[13] Coleman J.J. (2016). The *Fusarium solani* species complex: ubiquitous pathogens of agricultural importance. Mol. Plant Pathol..

[14] O'Donnell K., Sutton D.A., Fothergill A., McCarthy D., Rinaldi M.G., Brandt M.E. (2008). Molecular phylogenetic diversity, multilocus haplotype nomenclature, and *in vitro* antifungal resistance within the *Fusarium solani* species complex. J. Clin. Microbiol..

[15] Zhang N., O'Donnell K., Sutton D.A., Nalim F.A., Summerbell R.C., Padhye A.A. (2006). Members of the *Fusarium solani* species complex that cause infections in both humans and plants are common in the environment. J. Clin. Microbiol..

[16] Davis M.R., Poll C., Bonn W.V., Curtis E., Sattler M. (2007). Successful resolution of “bonnethead shark disease”, presumptive disseminated Fusarium infection, with antifungal therapy and environmental manipulation.

[17] Mylniczenko N., Clauss T., Smith Mark, Warmolts Doug, Thoney Dennis, Hueter Robert, Murray Michael, Ezcurra J. (2017). Pharmacology of elasmobranchs: updates and techniques. The Elasmobranch Husbandry Manual II: Recent Advances in the Care of Sharks, Rays and Their Relatives.

[18] Coleman J.J., Rounsley S.D., Rodriguez-Carres M., Kuo A., Wasmann C.C., Grimwood J. (2009). The genome of *Nectria haematococca*: contribution of supernumerary chromosomes to gene expansion. PLoS Genet..

